# Editorial

**Published:** 1841-01-23

**Authors:** 


THE MEDICAL EXAMINER.

PHILADELPHIA, JANUARY 23, 1841.

### ONTOLOGY.

The attacks of Broussais were directed with especial reference against the medical ontologists: these were often imaginary enemies, but giants, or wind-mills, they were the opponents with whom he was ever anxious for a tilt, if not for a sharper encounter. Still, the pure localization of disease would not suffice; he, too, was obliged to fall into ontology,—that is, to create abstractions, and to use them as links to bind together the structural lesions which he found, or believed he found, in all diseases. Hence he fell by the very weakness which he had spent the best years of his life in exposing; and after having shown that in disease the solids are all-important, he ended by reducing all disease to inflammation—the two terms becoming at last synonymous—dependent upon the vital irritability, or the doctrine of irritation.

This matter has been a subject of recent discussion at the French Academy of Medicine, and the speakers, Dubois, of Amiens, Rochoux, and others, for the most part, agreed that Broussais had attacked chimeras which had no existence but in his own brain, for no one actually believed in the separate existence of groups of symptoms which were classed together for convenience of description and study; it is true that the habit of studying these symptoms together, gives to each group an individual character which may lead to false reasoning and imperfect deduction. The method, however, is not the less necessary; and the attempt of Broussais to throw off the restraints of nosology, led him into much greater errors, and proved that although he rendered essential services to science as a severe and often a just critic of former errors, his mission was a limited one, and his works soon ceased to have any other than a pernicious effect on medicine.

This fault is not peculiar to Broussais; all reformers who attempt to go beyond their power, and to erect themselves into system makers, as well as critics, fail in the object of their ambition. They begin with a true principle like Broussais and Hahnemann, and end with generalizations, which are a mere play upon words, and an insult to common sense. There is no doubt, however, that medicine is susceptible of generalization; but the time is perhaps, not yet arrived, and he who attempts the task prematurely will be almost sure to fail. Perhaps no single individual will ever arrive at very great results, for it rarely happens that the discoveries of individuals do more than hasten the conclusion to which the labours of numbers are slowly arriving.

We cannot, therefore, get rid of ontology; in its restricted and legitimate application, it means merely, that certain sympathies occurring in a definite succession, receive a distinct appellation, and are regarded as an individual disease, whether the disorder of particular organs, or of the fluids of the economy, upon which these symptoms are supposed to depend, can be pointed out or not. There is no reasonable doubt that the disorders of the general structure of the body, or of the fluids, are equally definite as to the succession of symptoms, as those affections which are strictly local, but they are yet either unknown or imperfectly known. Hence, a true philosophy requires that these symptoms should, for the present at least, be regarded, not as the disease itself, but as collectively designating its nature, which may afterwards be rendered more definite by a knowledge of the organic changes which give rise to symptoms. If we keep this in view, and do not forget that the arrangement is provisional only, no possible error can result from it; on the contrary, it is the course which things naturally take, which in itself is a strong reason for believing it to be the correct one.

